# Introduction to the postanaesthetic care unit

**DOI:** 10.1186/2047-0525-2-5

**Published:** 2013-03-22

**Authors:** Joanna C Simpson, S Ramani Moonesinghe

**Affiliations:** 1 UCL Centre for Anaesthesia, University College Hospital, London, NW1 2BU, UK; 2 UCL/UCLH Surgical Outcomes Research Centre, University College Hospital, London, NW1 2BU, UK

**Keywords:** Anaesthesia recovery period, Perioperative care, Postoperative care

## Abstract

High-risk, noncardiac surgery represents only 12.5% of surgical procedures, but 83.3% of deaths. The postanaesthetic care unit (PACU) addresses the need for an improved level of care for these patients by providing postoperative high-dependency or intensive care (Level 2 or 3). The PACU aims to improve the structure of care provision for high-risk surgical patients. By maintaining 24-hour cover at the same staffing level, the risk of poorer ‘out-of- hours’ care is reduced. In a PACU, whose remit is solely postoperative care, evidence-based protocols can be established to standardize the care given. The aim is to provide 24 hours of postoperative optimized care, thus targeting the period when these patients are most vulnerable, to reduce the risk of complications developing and identify complications promptly, should they occur. The PACU is set up to facilitate certain processes to aid optimized care in the postoperative period. These include invasive and noninvasive ventilation, goal-directed haemodynamic management, invasive monitoring and optimal pain management. Identification of high-risk patients who might benefit from PACU care is not always straightforward. However, tools are available to aid the clinician, supplementing clinical assessment and basic investigations. These include clinical prediction rules and cardiopulmonary exercise testing. Both the setting up and the running of a PACU clearly have cost implications. However, the reduction in postoperative morbidity, and thus patients’ length of stay, should, overall, reduce costs. The benefits of a PACU should therefore be seen in terms of improved surgical outcomes, reducing postoperative morbidity and mortality, and cost savings.

## Introduction

High-risk noncardiac surgery is associated with significant morbidity and mortality rates. In one study, these high-risk cases represented 12.5% of surgical procedures, but 83.8% of deaths [1]. In the context of an aging population with an increased disease burden, it is likely that increasing numbers of high-risk surgical procedures will be performed [2].

A report from the National Confidential Enquiry into Patient Outcome and Death (NCEPOD) [3] concludes that there is a need for a system to identify patients with a high perioperative risk, and for improved postoperative care for these patients. A report by the Royal College of Surgeons in conjunction with the Department of Health [4] recommends that each patient should be assessed for risk at the end of surgery to help determine the optimal location for postoperative care and that those patients whose risk of death is calculated at ≥10% should be admitted to critical care. This report [4] goes on to recommend that National Health Service (NHS) Trusts should consider their spectrum of critical care provision and consider options for those at lower risk, to improve surgical outcomes further.

A recent cohort study, the European Surgical Outcomes Study (EuSOS), of over 46,000 patients undergoing noncardiac surgery in 28 European nations, showed that only 5% of cases underwent a planned admission to critical care, and those who had an unplanned admission to critical care had a higher mortality rate. Of the patients who died in this study, 73% were not admitted to critical care at all [5]. This suggests that throughout Europe there may be a systemic failure to provide critical care resources to those who need it in the perioperative period.

The postanaesthetic care unit (PACU) provides postoperative high-dependency or intensive care for high-risk surgical patients in an area separate from the general intensive care unit (ICU). It therefore addresses this well-established need for improved postoperative care for this population. It provides a clinical environment in which interventions aimed at improving outcomes, reducing morbidity and consequently length of stay, may be implemented. The concept of the PACU uses the example set to us by cardiac surgery where high-risk patients with multiple comorbidities undergo major surgery with a mortality of only 1.5% in 2008 [6] for coronary-artery bypass grafting. In this case, the low mortality for such high-risk surgery is likely to be at least partly attributable to the fact that all patients receive highly protocolized care, which includes routine admission to a Level 2 or 3 environment postoperatively.

In 1966, Donabedian looked at quality of healthcare in terms of ‘structure’, ‘process’ and ‘outcome’ [7]. Here, we define the PACU in terms of the processes it facilitates and the structure it provides. We will consider in each case how each of these elements will improve outcomes for patients undergoing high-risk surgery and how such ‘high-risk’ patients who might benefit from PACU care can be identified. Finally we will look at the financial justification for the implementation of a PACU system.

### How the postanaesthetic care unit improves the structure of care provision

#### High-intensity nursing and medical care

The PACU provides Level 2 and 3 care for high-risk surgical patients and is staffed by appropriately trained nurses. Each patient should be the joint responsibility of the surgical team and either an intensivist or anaesthetist.

Maintaining 24-hour cover at the same staffing level helps avoid variation in the standard of care and the potential for poorer care being delivered ‘out of hours’. It has been shown that for patients admitted to regular wards after nonemergency surgery, mortality is higher in those who have surgery on Friday than in those who have surgery between Monday and Wednesday [8]. However, in patients admitted to critical care postoperatively, no such difference in outcome was observed [8]. The increased ratio of nursing staff to patients, and the more reliable provision of ancillary services, such as physiotherapy, may contribute to an improved standard of care being given to these patients and consequently better outcomes. One study demonstrated an increased mortality for each additional patient per nurse [9].

‘Failure to rescue’, meaning death after a complication, is a concept that is gaining interest in research into surgical outcomes [10-15]. Comparison between American institutions shows no significant difference in complication rates, but a large range of mortality rates, that is, it was found that higher mortality institutions have higher ‘failure-to-rescue’ rates [11]. This suggests that prompt, appropriate management of postoperative complications is crucial to improving surgical outcome. Having a unit dedicated to the provision of care for postsurgical patients means that staff can be trained to focus on the issues that face this patient population, and therefore aim to reduce the incidence of ‘failure to rescue’ when complications arise.

#### Standardizing care

Practice varies between institutions [16] and outcomes vary both between institutions [11] and countries. The EuSOS demonstrated that even after adjustment for confounding factors, some countries had unexplained higher rates of perioperative mortality [5]. In a PACU with protocols designed and regularly updated by medical staff, practice can be brought in line with current best evidence, with the objective of optimizing surgical outcomes.

One study [17], in eight hospitals across the world with a variety of economic circumstances and diverse populations, found that the introduction of a surgical safety checklist resulted in a reduction in mortality rate from 1.5% to 0.8%. This shows that a simple intervention aimed at standardizing elements of perioperative care can have a significant impact, improving outcomes. A follow up multicentre study based solely in a high-income setting obtained similar results [18].

An example of a standardized, evidence-based package of care in which the PACU can play a significant role, is an enhanced recovery programme. The ratio of high nursing staff to patients in the PACU facilitates many of the elements key to enhanced recovery. These include goal-directed fluid therapy, epidural analgesia and early mobilization. The enhanced recovery programme calls for ‘a structured approach to immediate postoperative and during (perioperative) management, including pain relief’ [19], for which the PACU is ideally set up. Such enhanced recovery programmes have been shown, in colorectal surgery, to reduce postoperative morbidity and length of hospital stay [20-22]. There is also a growing body of evidence for their use in other surgical specialties, with one cohort study demonstrating reduced mortality in patients undergoing hip and knee replacements within an enhanced recovery programme compared with traditional care [22,23].

#### For whom and for how long?

The PACU targets those high-risk surgical patients who would otherwise be considered for postoperative critical care admission, thus taking pressure off the general ICU and removing competition for beds between emergency and postoperative admissions. The PACU will also take high-risk surgical patients, who in its absence might be sent to a general surgical ward postoperatively, thus providing these patients with all the additional facilities and staffing levels not available to them on a general surgical ward.

The PACU aims to provide nurse-led, protocol-driven care for up to 24 hours postoperatively, thus targeting the period when these high-risk surgical patients are most vulnerable prior to discharge to a surgical ward [24]. It is anticipated that by doing this, early complications will be recognized at the first opportunity and later complications might be prevented altogether. The PACU, therefore, should not need to deliver the advanced therapies, such as renal replacement therapy, that an ICU might offer [24], and it does not aim to replace the role of the ICU in providing prolonged periods of organ support.

### How the postanaesthetic care unit facilitates processes to improve care provision

#### Haemodynamic management

There is a large body of evidence to suggest that the perioperative use of goal-directed fluid therapy (meaning the use of cardiac output parameters to guide fluid and inotropic therapy) improves outcomes, reducing both complication rates and length of hospital stay [25-28]. A recently published systematic review and meta-analysis examining these interventions in higher-risk surgical patients has confirmed a mortality and morbidity benefit to haemodynamic optimization [29]. This is presumed to be due to the beneficial effect of better matching of oxygen supply to tissue oxygen consumption. The PACU provides an ideal setting for this postoperatively, with sufficient staff with appropriate levels of training to administer it.

The National Institute for Clinical Excellence recently issued guidelines recommending the use of the CardioQ-ODM (Deltex Medical) oesophageal Doppler device for in patients undergoing major or high-risk surgery, or other surgical patients for whom a clinician would consider using invasive cardiovascular monitoring. The PACU provides a suitable environment to continue such monitoring into the postoperative period. The oesophageal Doppler is not well tolerated by conscious patients. Therefore, for those who are extubated immediately postoperatively, other devices can be used, such as the LiDCO plus system [25], which relies on lithium dilution and pulse power analysis to derive cardiac output measurements.

#### Monitoring

The PACU allows continuous electrocardiography, which is particularly useful in those high-risk patients with pre-existing cardiac comorbidities, or in whom there were any intraoperative concerns about either rhythm disturbances or cardiac ischaemia. It also allows continuous oxygen saturation monitoring, which facilitates the titration of the inspired oxygen concentration to maintain optimal oxygen delivery. This is particularly useful in those patients at high risk of postoperative hypoxaemia, either because of the nature of the surgery (for example, abdominal surgery) or prescribed drugs (for example, high doses of opiates), or because of any comorbidities, such as chronic obstructive pulmonary disease.

The PACU provides a safe environment for more invasive monitoring postoperatively. This includes invasive arterial blood pressure monitoring, which is not usually possible on a standard general surgical ward. This, in turn, facilitates the safe use of inotropes in the perioperative period, should they be required. The intra-arterial catheter may also be used for cardiac output monitoring by pulse contour analysis and enables sampling of arterial blood to check blood gases, lactate levels, haemoglobin levels and electrolyte levels. The ability to measure these parameters will in turn enable clinicians to optimize ventilation and oxygen delivery, and recognize any developing complications at the earliest opportunity.

Clinical experience tells us that while central venous catheters may be used on a general surgical ward, they tend not to be commonplace. In the PACU, staff members are highly trained in their use, and safe maintenance. They can be used for central venous pressure monitoring and for obtaining mixed venous blood samples to assess oxygen saturation and hence help guide haemodynamic management.

#### Ventilation

There is evidence for the benefit of maintaining continuous positive airways pressure (CPAP) after elective major abdominal surgery [30]. This study showed that patients with postoperative hypoxaemia who received CPAP had a significantly reduced incidence of reintubation and pneumonia, as well as reduced lengths of stay in intensive care. The PACU provides an ideal environment for this, with adequate numbers of appropriately trained staff to give CPAP to those who need it. Clinical experience tells us that it is often difficult to facilitate this safely on a general surgical ward.

#### Pain management

Many patients having major surgery are fitted with an epidural catheter for perioperative pain management. The PACU is an excellent setting for the management of such devices, with nurses experienced in their use and anaesthetists readily available to deal with any problems that may arise.

The MASTER trial [31] concluded that most postoperative morbidities are not reduced by combined techniques using epidural and general anaesthesia. However, it does suggest that in view of the improved analgesia and reduction in respiratory failure, many high-risk patients undergoing major intra-abdominal surgery will receive substantial benefit from combined general and epidural anaesthesia intraoperatively with continuing postoperative epidural analgesia. These are precisely the patients who are also likely to require PACU care and will therefore also benefit from the improved epidural care in the PACU.

For patients receiving intravenous opiates, a PACU is again a good setting for the optimization of analgesia. With a higher ratio of nursing staff to patients, and continuous pulse oximetry and respiratory-rate monitoring, higher doses of intravenous opiates can be administered safely than would be possible on a general surgical ward with no continuous monitoring. In the PACU this may be via a patient controlled analgesia pump or, for sedated patients, via nurse-administered analgesia.

### Who are the high-risk surgical patients who might benefit from PACU?

It seems self-evident that the patients who will benefit most from a PACU are those whose surgery or comorbidities make them ‘high risk’. What is more difficult to ascertain is *how* ‘high risk’ a patient needs to be to benefit from care in the PACU, and how to identify these patients.

One study [1] grouped surgical procedures into healthcare resource groups (HRGs), many of which specify the presence of a complicating medical condition, the complexity of surgery or a particular age group. It defined ‘high risk’ as being a mortality rate of 5% or more and then assigned the HRGs to either a ‘high-risk’ or ‘low-risk’ category accordingly. This figure of 5% appears to be a useful cut-off: in this case, a high-risk population of 513,924 patients was identified (in which there were 63,340 deaths; 12.3%), this population accounted for 83.8% of deaths but only 12.5% of procedures. This study relied, however, on the coding being applied correctly, and brings into question how such terms as ‘complex comorbidities’ were defined.

The NCEPOD report [3] looked at surgery in March 2010 for 18,565 patients of whom the anaesthetists identified 3,734 (20%) as being high risk. In this report, the authors chose to ask the anaesthetist to decide whether the patient was high risk. In each case, the anaesthetists involved would have drawn upon their clinical experience and knowledge of the literature to make the risk assessments. This approach leaves room for variation in the criteria used to assess risk, and consequent variations in the management of such ‘high-risk’ patients. It also is likely to identify more patients than a PACU system can cope with; therefore a more refined process is required to assign different levels of risks to patients.

The report recognizes in its principal recommendations, therefore, that a UK-wide system should be introduced that allows rapid and easy identification of patients who are at high risk of postoperative mortality and morbidity.

Many approaches to such a quantification of perioperative risk have been made, and while none provide the perfect answer, it is likely that a combination of methods can provide a reasonable ‘best guess’. Perioperative risk and consequent postoperative outcomes are a result of the complex interplay between the exact general surgical procedure performed, the previous health of the patient, and specific intra- and postoperative events [32].

Much of this risk assessment does of course involve a thorough examination and basic investigations, as well as use of the patient’s history. However, interpretation of this information is operator dependent, so a number of other additional methods are used to standardize interpretation and provide extra information. This has been discussed in more detail [33], but some examples of these additional methods of risk assessment are given in Table 1.

**Table 1:** Standardized methods of risk assessment

**Method of risk assessment**	**Definition**	**Advantages**	**Limitations**
**Clinical prediction rules**	The use of a scoring system based on patient- or procedure-related risk factors to quantify risk	Often cost-neutral	Estimates population risk for patient rather than providing an individualized risk assessment
		Requires no specialist knowledge [34]	
**ASA-PS**	Six-point scale used to grade patient according to comorbidities [35]	Validated in a number of settings [36-38]	Inter-observer variability [39]
			Poor sensitivity and specificity for prediction of morbidity and mortality on an individual patient basis [33,40]
**Lee Revised Cardiac Risk Index (RCRI)**	Scores patients according to six variables, including whether the surgery is high risk	Discriminates moderately well between patients at low versus high risk for cardiac events after mixed noncardiac surgery [42]	Designed to identify patients at risk of cardiac complications so may miss patients at risk of other complications who may benefit from PACU care
	Assesses cardiac risk [41]	Well validated	
**POSSUM**	A more detailed scoring system with 18 components, 6 operative variables and 12 physiological variables [46]	A revision of POSSUM, the Portsmouth POSSUM [43] has been shown to be a better predictor of outcome in certain surgical settings [33,44,45]	Some variables cannot be ascertained until after surgery, making it of limited use for preoperative identification of patients who may benefit from PACU care
		Variations in the model have been devised for specific patient groups, such as the Cr-POSSUM (colorectal), which has been shown to be a better predictor of outcome in this type of surgery [47]	
**Cardiopulmonary exercise testing**	CPET is an integrative and quantitative measure of a patient’s cardiopulmonary reserve	Good evidence that CPET is useful to help predict perioperative morbidity and mortality and may aid triage to an appropriate level of postoperative care [48-50]	In 2008, 17% of Hospital Trusts in England had a CPET service, and a further 7% were in the process of setting one up [52]
	The assessment requires the patient to exercise (usually on a cycle ergometer) while oxygen consumption, carbon dioxide production, and other cardiorespiratory variables are measured	RCT in progress to further evaluate its use to stratify to appropriate level of postoperative care [51]	

ASA-PS, American Society of Anesthesiology Physical Status Score; CPET, cardiopulmonary exercise testing; PACU, postanaesthetic care unit; POSSUM, physiological and operative severity score for the enumeration of mortality and morbidity; RCRI, Lee Revised Cardiac Risk Index; RCT, randomized controlled trial.

### How might this be implemented in your institution?

The NCEPOD report [3] indicates that NHS Trusts should plan to fund critical care facilities for postoperative care of high-risk surgical patients. One of its key recommendations is that the volume of high-risk work should be analyzed, to quantify the critical care requirements of this cohort, and the results should be reported to the Trust Board annually.

The implementation of such postoperative care facilities does have a significant cost implication, which is of particular relevance in the current financial climate. There is likely to be an initial cost to set up an appropriate area for the PACU and obtain the equipment it will require. Depending on the individual institution, much of this may be available if an area of a pre-existing ICU or recovery area can be used for the PACU. Ongoing costs will also be generated by the high staffing levels, and use of equipment.

However, the PACU should reduce costs overall, by reducing postoperative morbidity and consequently reducing patients’ length of stay in hospital. This is highlighted in a report by the Improving Surgical Outcomes Group (ISOG) [53]. This report considers possible improvements to perioperative care, including appropriate triaging of patients to postoperative critical care facilities (such as a PACU), resulting in patients, ‘leaving hospital faster and fitter following surgery, with the corresponding financial savings and service capacity benefits’ [53]. This report cited data from the Intensive Care National Audit and Research Centre (ICNARC), which shows that the mortality rate is higher among patients who are transferred from surgery to a general ward and then to the ICU than it is for those transferred directly to the ICU (42.5% vs. 19.9%), and that the total length of hospital stay is reduced in those patients transferred directly to the ICU [53]. When considering cost-effectiveness, reductions in length of stay play a crucial role.

The PACU may benefit from being physically separate from the ICU, as in general it cares for patients requiring less support than the ICU. This is likely to result in a better environment for patients recovering from surgery, with less noise, light and patient care interactions at night. This is likely to promote better sleep and thus aid recovery [54]. However, it is important to be pragmatic, and in many cases it may be more cost-effective and practical to have a PACU that is part of the ICU. This should not affect the structures and processes brought by a PACU that improve outcome. Individual institutions setting up such a system would need to ensure that competition for beds by other emergency patients did not result in perioperative patients missing out on PACU care.

### The international context

The evidence for improving outcomes, with its consequent cost-saving, can be seen globally. A study comparing a centre in the USA with one in the UK demonstrated a fourfold higher mortality in the UK [55]. While many factors may account for this, it is striking that in the USA there are considerably more critical care beds per capita than in the UK (20.0 compared to 3.5 per 100,000 people [56]). Perhaps this results in a better standard of care. Another study suggests that improved outcomes in the USA compared with the UK may not only be the consequence of the larger amount of money spent per capita. In one study [57], a Californian hospital achieved better performance at similar costs to the NHS.

Similarly, in Germany there is a reported sevenfold greater provision of critical care beds than in the United Kingdom [56] and there is a correspondingly higher postoperative critical care admission rate and a lower perioperative hospital mortality [5]. It is possible that the greater provision of postoperative critical care in Germany contributes to these superior mortality figures.

The EuSOS clearly demonstrates that across Europe there is a need for greater critical care provision for patients postoperatively. The majority of patients who died (73%) in this study were not admitted to critical care at any stage following surgery. Unplanned admissions to critical care in this cohort were associated with a higher mortality than planned admissions [5]. This need for higher intensity care postoperatively could be met by PACUs.

## Conclusion

Postoperative morbidity and mortality among high-risk patients is high, and a significant burden on healthcare resources. The PACU aims to improve the structure and facilitate the processes essential to provide the best-quality, evidence-based postoperative care. Identifying those high-risk patients who may benefit from such care remains difficult, and we will always fall short of predicting the future. However, clinical assessment, aided by clinical prediction rules and measurement of cardiopulmonary function should help determine which patients may benefit from PACU care. A PACU should, in the long-term, improve the quality of patient care, reducing the burden of postoperative morbidity both for the individual patients and for the purses of the institutions caring for them.

## Abbreviations

ASA-PS: American Society of Anesthesiology Physical Status Score; CPAP: Continuous positive airways pressure; CPET: Cardiopulmonary exercise testing; HRG: Healthcare resource group; ICNARC: Intensive Care National Audit and Research Centre; ICU: Intensive care unit; ISOG: Improving Surgical Outcomes Group; NCEPOD: National Confidential Enquiry Into Patient Outcome and Death; NHS: National Health Service; PACU: Postanaesthetic care unit; POSSUM: Physiological and operative severity score for the enumeration of mortality and morbidity; RCRI: Lee Revised Cardiac Risk Index; RCT: Randomized controlled trial

## Competing interests

The authors declared that they have no competing interests.

## Authors’ contributions

JS drafted the manuscript. SRM helped draft the manuscript. Both authors read and approved the final version.

## Authors’ information

SRM works within the UCLH/UCL Joint Comprehensive Biomedical Research Centre, which receives funding from the UK Department of Health’s National Institute for Health Research Centres’ funding scheme.
